# Survey on chest CT findings in COVID-19 patients in Okinawa, Japan: differences between the delta and omicron variants

**DOI:** 10.1038/s41598-023-47756-8

**Published:** 2023-11-21

**Authors:** Nanae Tsuchiya, Eri Yonamine, Shoko Iraha, Makoto Takara, Yasuji Oshiro, Miyara Tetsuhiro, Sadayuki Murayama, Ryo Kinoshita, Masaki Sato, Yukiko Nishikuramori, Hiroaki Takara, Tamaki Akamine, Hikaru Morita, Takashi Matayoshi, Yuma Chinen, Akihiro Nishie

**Affiliations:** 1 Department of Radiology, Graduate School of Medical Science, University of the Ryukyus, 207 Uehara, Nishihara-cho, Nakagami-Gun, Okinawa, 903-0215 Japan; 2 Department of Radiology, Okinawa Kyodo Hospital, Okinawa, Japan; 3 Department of Radiology, Heart Life Hospital, Okinawa, Japan; 4 Department of Radiology, National Hospital Organization, Okinawa National Hospital, Okinawa, Japan; 5 Department of Radiology, Urasoe General Hospital, Okinawa, Japan; 6 Department of Radiology, Okinawa Prefectural Nanbu Medical Center and Children’s Medical Center, Okinawa, Japan; 7 Department of Radiology, Nakagami Hospital, Okinawa, Japan; 8 Department of Radiology, Okinawa Chubu Hospital, Okinawa, Japan; 9 Department of Radiology, Yuai Medical Center, Okinawa, Japan; 10 Department of Radiology, Ohama Daiichi Hospital, Okinawa, Japan; 11 Department of Radiology, Naha City Hospital, Okinawa, Japan

**Keywords:** Diseases, Medical research

## Abstract

To investigate the frequency of pneumonia and chest computed tomography (CT) findings in patients with coronavirus disease 2019 (COVID-19) during the fifth Delta variant-predominant and sixth Omicron variant-predominant waves of the COVID-19 pandemic in Okinawa, Japan. A survey on chest CT examinations for patients with COVID-19 was conducted byhospitals with board-certified radiologists who provided treatment for COVID-19 pneumonia in Okinawa Prefecture. Data from 11 facilities were investigated. Indications for chest CT; number of COVID-19 patients undergoing chest CT; number of patients with late-onset pneumonia, tracheal intubation, and number of deaths; and COVID-19 Reporting and Data System classifications of initial chest CT scans were compared by the chi-squared test between the two pandemic waves (Delta vs. Omicron variants). A total of 1944 CT scans were performed during the fifth wave, and 1178 were performed during the sixth wave. CT implementation rates, which were the number of patients with COVID-19 undergoing CT examinations divided by the total number of COVID-19 cases in Okinawa Prefecture during the waves, were 7.1% for the fifth wave and 2.1% for the sixth wave. The rates of tracheal intubation and mortality were higher in the fifth wave. Differences between the distributions of the CO-RADS classifications were statistically significant for the fifth and sixth waves (*p* < 0.0001). In the fifth wave, CO-RADS 5 (typical of COVID-19) was most common (65%); in the sixth wave, CO-RADS 1 (no findings of pneumonia) was most common (50%). The finding of “typical for other infection but not COVID-19” was more frequent in the sixth than in the fifth wave (13.6% vs. 1.9%, respectively). The frequencies of pneumonia and typical CT findings were higher in the fifth Delta variant-predominant wave, and nontypical CT findings were more frequent in the sixth Omicron variant-predominant wave of the COVID-19 pandemic in Okinawa, Japan.

## Introduction

Since the beginning of the coronavirus disease 2019 (COVID-19) pandemic, multiple variants of severe acute respiratory syndrome coronavirus 2 (SARS-CoV-2) have been identified worldwide. These variants have different degrees of transmissibility and disease severity^1^. Multiple waves of infection have occurred in Okinawa Prefecture because of the emergence of mutated strains. After the fifth wave of infection, in which the Delta variant was predominant, Okinawa entered the sixth wave, in which the Omicron variant was predominant. Okinawa Prefecture had the highest cumulative number of infected people per 100,000 people in Japan in these two waves.

The Delta variant was first isolated in India in December 2020 and was 50% more transmissible than the Alpha variant^1^. In Japan, the Delta variant was first detected in March 2021 at an airport quarantine station, and a domestic infection was confirmed in April 2021. The variant then spread throughout the country. Replacement of the Alpha variant by the Delta variant progressed rapidly; approximately 90% of the isolates’ genomes that were decoded from patient samples collected in August 2021 from metropolitan areas were identified as the Delta variant.

The National Institute of Infectious Diseases (NIID) used the haplotype network to determine that there were at least 7 strains causing COVID-19 in Japan that originated abroad^2^. During the early days of the epidemic of the Delta strain, 6 of the 7 strains disappeared, and the remaining Delta strain subsequently spread nationwide^2^. In Okinawa, the Delta variant was first detected on June 24, 2021, and it became the mainstream strain, accounting for 90% of infections in approximately 1 month. The Delta variant has been associated with an increased risk of hospitalization and mortality^1^.

The Omicron variant was first isolated in South Africa on November 24, 2021, became prevalent in more than 90 countries, and was the predominant strain in the United States of America, with more than 50 mutations in the spike protein. In Japan, the Omicron variant was first detected on November 30, 2021, at an airport quarantine station. Subsequently, reports of detection of the Omicron variant were issued from multiple prefectures within a single month. Approximately 10% of these variants were isolated from infected patients without a history of travel out of the country^3^.

In Okinawa, the Omicron variant was first detected on December 17, 2021, at Camp Hansen, which is a United States Marine Corps base. In approximately 2 weeks, the Omicron variant accounted for 90% of infections in Okinawa. This Omicron variant remains the variant of greatest concern^1,4,5^. The Omicron variant appears to be associated with decreased severity of disease and a decreased risk of hospitalization^1^.

Chest computed tomography (CT) is widely used to detect COVID-19 pneumonia, assess its severity, and estimate the prognosis of patients^1,6^. It has been reported that the lung damage caused by the Omicron variant is generally milder than the damage due to other variants. Chest CT scans of patients infected with the Omicron variant show a higher prevalence of nontypical findings such as bronchopneumonia/bronchiolitis than chest CT scans of patients infected with previous mainstream variants^7–11^. Therefore, to avoid mistaken clinical assessments of CT scans that are based on conventional criteria, it is important to investigate the changes in imaging findings in patients infected with variants of SARS-CoV-2.

The purpose of this study was to investigate the frequency of pneumonia and chest CT findings in COVID-19 patients during the fifth wave of the COVID-19 pandemic, in which the Delta variant was predominant, and the sixth wave, in which the Omicron variant was predominant, in Okinawa, Japan.

## Materials and methods

This research was conducted in compliance with the “Declaration of Helsinki (revised in October 2013)” and the “Ethical Guidelines for Life Science and Medical Research Involving Human Subjects (Ministry of Education, Culture, Sports, Science and Technology, Ministry of Health, Labour and Welfare, Ministry of Economy, Trade and Industry Notification No. 1 in Reiwa 3)”.We performed a survey of chest CT examinations of patients with COVID-19 at 13 hospitals located in the central and southern areas of Okinawa Prefecture. These hospitals have board-certified radiologists and provide inpatient treatment for patients with COVID-19 pneumonia. Hospitals in the outlying islands (Miyako and Yaeyama Islands) and northern districts were excluded because of differences between the duration of waves of the COVID-19 pandemic in the islands and northern districts versus the waves in the central and southern areas of Okinawa Prefecture. We summarized the responses of 11 hospitals with complete answers.

### Definition of pandemic wave

**Figure 1:**
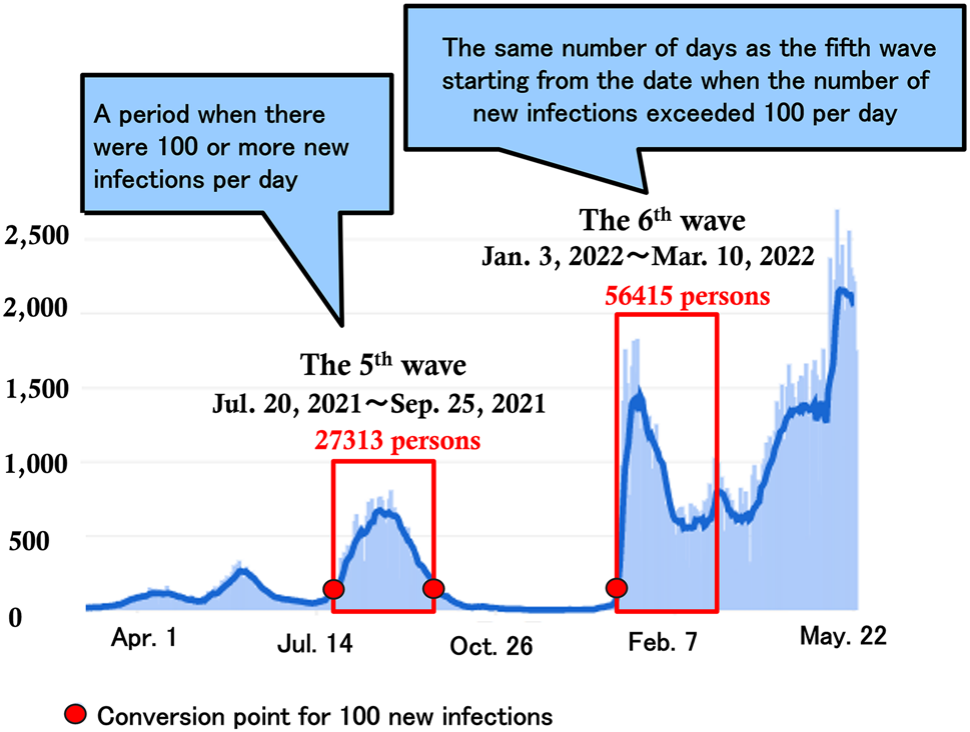
Definition of pandemic wave. The fifth wave of the pandemic was defined as a period when there were 100 or more new infections per day. For the sixth wave, the survey was conducted for the same number of days as the fifth wave starting from the date when the number of new infections exceeded 100 per day.

We defined the fifth wave of the pandemic as a period when there were 100 or more new infections per day (July 20 to September 25, 2021). For the sixth wave, the survey was conducted for the same number of days as the fifth wave (January 3 to March 10, 2022), starting from the date when the number of new infections exceeded 100 per day (Fig. 1). The total population of Okinawa Prefecture was 1,459,214 in July 2021. According to the reports of the Okinawa Prefecture COVID-19 Control, Epidemiology, and Statistical Analysis Committee, the proportion of Delta variant-caused infections was 78.9% during the week starting July 27, 2021, and the proportion of Omicron variant-caused infections was 87.2% during the week starting January 3, 2022. Therefore, the Delta variant was predominant during the fifth wave, and the Omicron variant was predominant in the sixth wave in Okinawa, Japan. Table 1 shows the data from the Okinawa Prefecture COVID-19 Control, Epidemiology, and Statistical Analysis Committee^5^.

**Table 1:** Data from the Okinawa Prefecture COVID-19 control, epidemiology, and statistical analysis committee^5^.

Pandemic wave	5th Wave (Delta predominant)		6th Wave (Omicron predominant)	
Duration (Dates of initiation-termination)	Jul. 20, 2021	Sep. 25, 2021	Jan. 3, 2022	Mar. 10, 2022
Number of new cases	154	115	130	649
Total number of cases during the study period	27,313		56,415	
Deaths	77 (0.28%)		32 (0.06%)	
Variants	Delta 78.9%^a^		Omicron 87.2%^b^	
Vaccination rate	Jul. 16, 2021	Sep. 24, 2021	Jan. 3, 2022	Mar. 15, 2022
Single (%)	21.8	60.4	69.9	70.7
Double (%)	12.2	47.1	68.9	69.9
Booster (%)	0	0	0.8	24.7

*COVID-19* coronavirus disease 2019.

^a^Determined week of Jul. 27, 2021. ^b^Determined week of Jan. 3, 2022.

### Survey

The list of survey questions was as follows:

Question 1: Chest CT criteria for COVID-19 patients.

The 3 criteria of patients with COVID-19 for undergoing chest CT were as follows: (1) Positive result on a reverse-transcriptase polymerase chain reaction [RT‒PCR] assay for SARS-CoV-2; (2) high-risk or hospitalized patients with moderate to severe COVID-19; and (3) determination by physicians.

Question 2: Number of patients with COVID-19 (confirmed by an RT‒PCR assay for SARS-CoV-2, genetic testing results were not required) who underwent chest CT during each wave (repeated exams for the same patient were not counted; only the initial CT assessment was counted).

Question 3-1 (for the cohort of Question 2): Characteristics of patients with COVID-19 who underwent CT:

Age (mean, median, minimum, maximum), percentage of male patients, number of patients needing tracheal intubation, number of patients who died, and number of patients with late-onset pneumonia who were classified as COVID-19 Reporting and Data System(CO-RADS) 1 at the initial assessment and had pneumonia on a repeated CT examination.

Question 3-2 (for the cohort of Question 2): CT image classification.

Number of patients in each COVID-19 Reporting and Data System CT image category (CO-RADS 0–5, Table 2).

**Table 2:** Chest CT findings of CO-RADS classification.

CO-RADS	Judgment	Imaging findings
Category 0 Not interpretable	Scan technically insufficient for assigning a score	Severe artifacts due to coughing or breathing
Category 1 Very Low	Normal or noninfectious	Negative for pneumonia (includes baseline lung disease, i.e., tumor, emphysema)
Category 2 Low	Typical for other infections but not COVID-19	Tree-in-bud sign, a centrilobular nodular pattern, lobar or segmental consolidation, lung cavitation
Category 3 Equivocal/unsure	Features compatible with COVID-19 but also other diseases	Perihilar GGO, homogenous extensive GGO with or without sparing of some secondary pulmonary lobules, or GGO together with smooth interlobular septal thickening with or without pleural effusion in the absence of other typical CT findings
Category 4 High	Suspicious for COVID-19	Typical for COVID-19 but also showing some overlap with other (viral) pneumonia: not in contact with the visceral pleura, located strictly unilaterally in a predominantly peribronchovascular distribution, or superimposed on severe diffuse preexisting pulmonary abnormalities
Category 5 Very High	Typical for COVID-19	GGO with or without consolidations, in lung regions close to visceral pleural surfaces, including the fissures (subpleural sparing is allowed) and multifocal bilateral distribution Ground-glass regions (unsharp demarcation, rounded shape, sharp demarcation, outlining the shape of multiple adjacent secondary pulmonary lobules), crazy paving, patterns compatible with organizing pneumonia, thickened vessels within parenchymal abnormalities found in all confirmatory patterns

*CT* computed tomography, *CO-RADS* COVID-19 reporting and data system, *COVID-19* coronavirus disease 2019, *GGO* ground-glass opacity.

### CT image classification

Chest CT images were classified into 5 categories according to CO-RADS (Table 2). The categories express the level of suspicion for COVID-19 pneumonia^6^ as follows: CO-RADS category 0, not interpretable (scan technically insufficient for assigning a score); CO-RADS category 1, very low (normal or noninfectious); CO-RADS category 2, low (typical for other infection but not COVID-19); CO-RADS category 3, equivocal/unsure (features compatible with COVID-19 pneumonia but also other diseases); CO-RADS category 4, high (suspicious for COVID-19 pneumonia); and CO-RADS category 5, very high (typical for COVID-19 pneumonia). CT classifications were performed by experienced radiologists at each institution.

### Statistical analysis

Patients’ ages are expressed as the means and were compared by the paired t test. Categorical variables are expressed as numbers and percentages, and the variables were compared between the 2 waves by the chi-squared test. The CT implementation rate, which was the number of patients with COVID-19 undergoing CT examinations divided by the total number of COVID-19 cases in Okinawa Prefecture during the waves, was calculated. JMP 11 (SAS Institute Japan, Tokyo, Japan) was used for statistical analysis. A p value less than 0.05 was considered statistically significant.

### Ethics approval

This survey was approved by the Ethics Committee for Clinical Research of University of the Ryukyus (approved number 23-2197-00-00-00). Written informed consent was obtained from all the participating facilities.

## Results

Question 1. In the fifth wave, the CT criterion of PCR positivity was most frequently selected (5/11 institutions, 45%). In the sixth wave, the CT criterion of high-risk or hospitalized patients with moderate to severe disease was most frequently selected (5/11 institutions, 45%). A change between the criteria in the two waves was made at 3 institutions (Tables 3, 4).

**Table 3:** Chest CT criteria for COVID-19 patients.

	5th Wave (Delta predominant)	6th Wave (Omicron predominant)
(1) All patients with PCR positivity	5 (45%)	3 (27%)
(2) High-risk/hospitalized patients with moderate to severe disease	4 (36%)	5 (45%)
(3) Determined by physicians	2 (18%)	3 (27%)

*CT* computed tomography, *COVID-19* coronavirus disease 2019, *PCR* polymerase chain reaction.

**Table 4:** CT criteria and number of patients who underwent CT at each institution.

Institutions	5th Wave (Delta predominant)		6th Wave (Omicron predominant)	
Hp1	163	(1)	70	(2)
Hp2	66	(2)	62	(3)
Hp3	184	(2)	145	(2)
Hp4	219	(2)	111	(2)
Hp5	223	(1)	138	(1)
Hp6	128	(1)	80	(1)
Hp7	249	(1)	90	(1)
Hp8	240	(2)	104	(2)
Hp9	244	(3)	132	(3)
Hp10	150	(3)	142	(3)
Hp11	78	(1)	103	(2)
Total	1944		1177	
Percentage*	7.1%		2.1%	

*CT* computed tomography, *Hp* hospital.

(1) All patients with PCR positivity, (2) High-risk/hospitalized patients with moderate to severe disease, (3) Determination by physicians.

*Percentage = total CT cases/total cases of COVID-19 during the duration of the study in Okinawa, Japan.

Question 2. The total numbers of patients with COVID-19 who underwent chest CT in the fifth Delta-predominant and sixth Omicron-predominant waves were 1944 and 1178 patients, respectively. The CT implementation rates were 7.1% for the fifth wave and 2.1% for the sixth wave (Table 4).

Question 3-1. The characteristics of COVID-19 patients undergoing chest CT are summarized in Table 5. Patients in the sixth Omicron-predominant wave were older than patients in the fifth Delta-predominant wave. Tracheal intubation and mortality rates were higher for the fifth Delta-predominant wave than for the sixth Omicron-predominant wave (intubation, 4% vs. 1.2%, respectively; *p* < 0.001) (mortality, 1.4% vs. 0.7%, respectively; *p* < 0.045), and rates were higher in the fifth wave (Delta predominant). Differences between distributions of the sexes of patients (percentage of males, 55% vs. 52%; *p* = 0.08) and the rates of late-onset pneumonia (3.0% vs. 1.3%; *p* = 0.2) were not significant between the two waves (Table 5).

**Table 5:** Characteristics of COVID-19 patients who underwent CT.

		5th Wave (Delta) N = 1944	6th Wave (Omicron) N = 1177	*p* Value
Age	Mean	52	63	0.0009*
	Median	50	62	
	Minimum	0	4	
	Maximum	102	103	
Sex	Men	1074 (55%)	611 (52%)	0.08
	Women	870 (45%)	566 (48%)	
	Tracheal intubation	78 (4.0%)	14 (1.2%)	< 0.001*
	Death	28 (1.4%)	8 (0.7%)	0.045*
	Late-onset pneumonia	36 (3.0%)	15 (1.3%)	0.2

*CT* computed tomography, *COVID-19* coronavirus disease 2019.

*p < 0.05 considered statistically significant.

**Figure 2:**
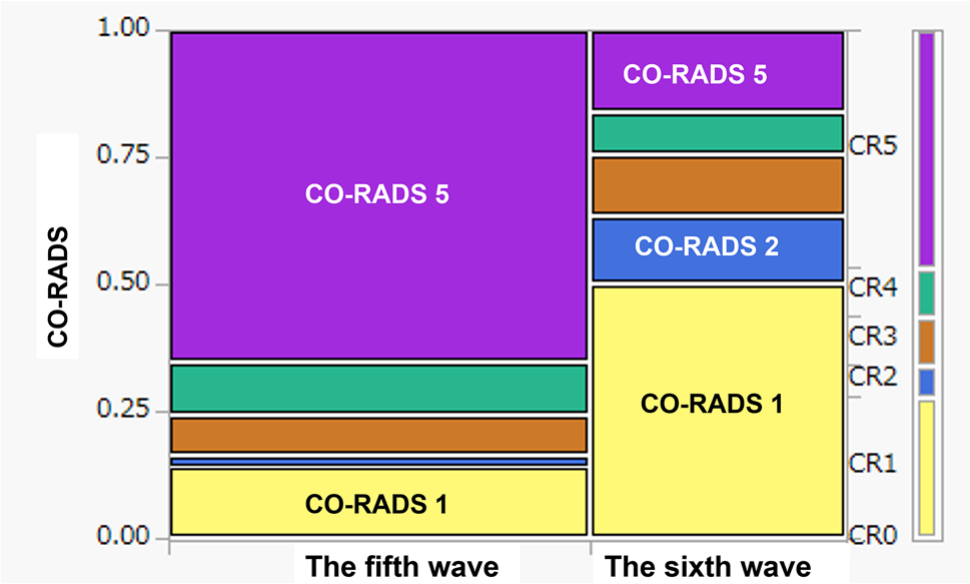
Diagram of CO-RADS classification during the pandemic waves. During the fifth wave, CO-RADS 5 (typical for COVID-19) was most common, and CO-RADS 1 (normal or noninfectious) was less common, whereas during the sixth wave, CO-RADS 1 was most common, and CO-RADS 5 was less common. CO-RADS 2 (typical for other infections but not COVID-19) was more frequent in the sixth wave than in the fifth wave.

Question 3-2: There were significant differences between the distributions of patients in specific CO-RADS categories for the patients in the fifth Delta-predominant wave and the patients in the sixth Omicron-predominant wave (*p* < 0.0001). In the fifth wave, most patients were classified into CO-RADS 5 (65.3%, 1270/1944), and fewer were classified into CO-RADS 1 (14.3%; 279/1944). In the sixth wave, most patients were classified into CO-RADS 1 (50%, 589/1177), and fewer were classified into CO-RADS 5 (16.1%, 189/1177). The sixth wave had more patients in CO-RADS 2 than the fifth wave (13.6% [160/1944] vs. 1.9% [37/1177], respectively). Two patients in the fifth wave were classified into CO-RADS 0 because of motion artifacts (failed breath-holding) in the fifth wave. Differences between the distributions of the patients in the fifth and sixth waves in CO-RADS 3 (1.9%, 37/1944 vs. 13.6%, 160/1177, respectively) and CO-RADS 4 (8.5%, 100/1944 vs. 10.1%, 197/1177) were not significant (Table 6, Fig. 2).

**Table 6:** Chest CT classifications of COVID-19 patients.

CO-RADS	5th Wave (Delta predominant) N = 1944	6th Wave (Omicron predominant) N = 1177
Category 0	2 (0.001%)	0 (0%)
Category 1	279 (14.3%)	589 (50%)
Category 2	37 (1.9%)	160 (13.6%)
Category 3	159 (8.2%)	139 (11.8%)
Category 4	197 (10.1%)	100 (8.5%)
Category 5	1270 (65.3%)	189 (16.1%)

*CT* computed tomography, *CO-RADS* COVID-19 reporting and data system, *COVID-19* coronavirus disease 2019.

## Discussion

In this study, changes between the indications for CT were made at 3 centers during 2 waves of the pandemic. During the sixth Omicron-predominant wave, there was a change to more limited indications for CT compared to the fifth Delta-predominant wave, and none of the centers increased their indications for CT. One possible reason for the change was that the number of infected people increased during the sixth wave. Therefore, it was not feasible to require that every patient undergo a chest CT examination, which would include patients who were asymptomatic or had mild symptoms. Another possible reason was that, based on the CT findings of the patients in the fifth wave, the importance of chest CT for mildly ill patients was low; therefore, it was decided to reduce the number of indications for chest CT during the sixth wave. Accordingly, the number of patients in the sixth wave who underwent CT was lower than the number of patients who underwent CT in the fifth wave. In the sixth wave, the proportion of patients undergoing CT decreased, which reflects the characteristics of the Omicron variant, which is more infectious but less severe than the Delta variant.

A comparison between patients in the fifth and sixth waves who underwent CT showed that the rates of tracheal intubation and mortality were higher in the fifth wave than in the sixth wave, indicating that patients infected with the Delta variant were more likely to develop severe disease than patients infected with the Omicron variant. Other prefectures reported results similar to ours, namely, that more patients among the total number of infected patients died in the fifth wave than in the sixth wave^5^.

This study found that in Okinawa, Japan, the frequencies of pneumonia and typical chest CT findings in COVID-19 patients were higher in the Delta-predominant pandemic wave than in the Omicron-predominant wave and that the frequency of nontypical CT findings was more frequent in the Omicron-predominant pandemic wave than in the Delta-predominant pandemic wave.

In the fifth Delta-predominant wave, CO-RADS 5 was the most common classification of a chest CT scan, while in the sixth Omicron-predominant wave, CO-RADS 1 was the most common classification. The distribution of CO-RADS classifications changed between the 2 pandemic periods. This finding suggests that the frequency of COVID-19 pneumonia decreased during the Omicron-predominant wave. A Chinese study reported a rate of 1.3% for pneumonia associated with the Omicron-predominant wave^8^. Tsakok et al. reported that the frequency of pneumonia differs between Delta and Omicron variants depending on vaccination status. With the Delta variant, the frequency of pneumonia complications decreases as the number of vaccinations increases, such as no vaccine, one or two doses, and booster doses. On the other hand, Omicron has the lowest incidence of pneumonia after single or double doses^10^. In our study, 0% of patients completed the booster dose in the fifth Delta-predominant wave, and there was a high rate of single or double doses in the vaccinated population in the sixth Omicron-predominant wave. This vaccination rate may be related to the lower frequency of pneumonia in the sixth wave.

Although the rate of pneumonia during the Omicron-predominant wave was lower than the rate during the Delta-predominant wave, it is important to understand that COVID-19 pneumonia associated with the Omicron variant can be typically severe and result in death^12^. It has been reported that there was no difference between the extent of the severity of pneumonia in vaccinated and unvaccinated ICU patients and in patients with pneumonia associated with the Omicron variant or Delta variant^13^. The rates of severity of pneumonia associated with the Omicron and Delta variants have been different, but the extents of the severity of the pneumonia associated with the 2 variants have been the same in severely ill patients.

During the sixth Omicron-predominant wave, the proportion of patients with CT scans classified as CO-RADS 2, which is considered to be an infection other than COVID-19, increased. Reports from South Korea^7^ showed that the Omicron variant manifested the typical CT appearance of COVID-19 pneumonia less frequently than the Delta variant (32% vs. 57%, respectively) and manifested a CT appearance of peribronchovascular pneumonia more frequently than the Delta variant (38% vs. 7%, respectively). When adjusted for age, comorbidities, vaccination status, and duration of infection, patients infected with the Omicron variant who developed pneumonia were found more frequently to manifest atypical peribronchovascular pneumonia than patients infected with the Delta variant who developed pneumonia. It has been thought that the Omicron variant is limited to the upper respiratory tract, with less involvement of the lungs. It has also been speculated that the number of COVID-19 patients who developed pneumonia (aspiration pneumonia, bacterial pneumonia, other viral pneumonia) other than COVID-19 pneumonia increased during the Omicron infection period. It has been reported that the complication of fungal/bacterial pneumonia in patients with COVID-19 pneumonia occurred in 8%, and the complication of other viral infections occurred in 20.7%^1^. Further consideration is needed.

This study has limitations. First, the actual SARS-CoV-2 variants that infected the patients in this study were not confirmed by genomic analysis. Second, the differences between the number of cases and the indications for CT examinations in the two waves may have biased the results. Third, the CO-RAD classifications might have been inconsistent because the CT scans were assessed by different radiologists at each hospital. Finally, the patients were not investigated for concomitant bacterial or other viral pneumonia.

In conclusion, in Okinawa, Japan, the frequencies of pneumonia and typical chest CT findings were higher during the fifth Delta variant-predominant wave, and nontypical CT findings that indicated infections other than COVID-19 pneumonia were more frequent during the sixth Omicron variant-predominant wave of the COVID-19 pandemic. It is important to recognize that the emergence of new variants not only changes the transmissibility of the virus and disease severity but also changes characteristic imaging findings. Radiologists should be aware that they should exercise caution in applying conventional criteria for evaluations of CT scans in patients infected with new variants causing COVID-19 pneumonia.

## Data Availability

The datasets used and/or analysed during the current study available from the corresponding author on reasonable request.
